# m^6^A-modified circARHGAP12 promotes the aerobic glycolysis of doxorubicin-resistance osteosarcoma by targeting c-Myc

**DOI:** 10.1186/s13018-023-04502-0

**Published:** 2024-01-04

**Authors:** Dawei Zhang, Qingzhu Guo, Kemin You, Yi Zhang, Yao Zheng, Tao Wei

**Affiliations:** 1 Department of Orthopedics, Zibo Central Hospital, Shandong First Medical University, Zibo, 255036 Shandong China; 2 Department of Emergency, Zibo Central Hospital, Shandong First Medical University, Zibo, 255036 Shandong China; 3 Department of Pathology, Zibo Central Hospital, Shandong First Medical University, Zibo, 255036 Shandong China

**Keywords:** Osteosarcoma, circARHGAP12, Doxorubicin, Aerobic glycolysis, c-Myc

## Abstract

Chemotherapy resistance accompanied by energy metabolism abnormality functions as one of the main reasons for treatment failure and poor prognosis. However, the function of N^6^-methyladenosine (m^6^A)-modified circular RNA (circRNA) on osteosarcoma (OS) is still unclear. Here, present research investigated the potential role and mechanism of circARHGAP12 on OS doxorubicin (Dox) resistance and aerobic glycolysis. Results indicated that circARHGAP12 was a novel m^6^A-modified circRNA, which was up-regulated in OS cells. Overexpression of circARHGAP12 promoted the Dox resistance half-maximal inhibitory concentration (IC50) and aerobic glycolysis (glucose uptake, lactate and ATP production) in OS cells (Saos-2/Dox, MG63/Dox). Mechanistically, m^6^A-modified circARHGAP12 could bind with c-Myc mRNA through m^6^A-dependent manner, thereby enhancing the c-Myc mRNA stability. Thus, these findings revealed the critical function of circARHGAP12 on OS Dox-resistance and aerobic glycolysis. Taken together, our study demonstrated a critical function of circARHGAP12 on OS chemotherapy resistance and energy metabolism abnormality, providing critical roles on OS treatment.

**Supplementary Information:**

The online version contains supplementary material available at 10.1186/s13018-023-04502-0.

## Introduction

Osteosarcoma (OS) is characterized by the most frequent primary solid malignancy in bone, with exceptional high incidence in young individuals or children [1, 2]. Despite the great improvements of OS therapeutic methods, including surgery incorporated with chemoradiotherapy [3], the five-year OS survival rate is approximate 70% (for localized type) and approximate 30% (recurrent/metastatic type) [4]. Thus, a better accurate target and novel understanding of OS molecular mechanism is critical for OS therapeutic strategies development.

Doxorubicin (DOX) chemotherapy integrated with cisplatin acts as the clinical standard treatment for OS patients [5]. Nevertheless, the resistance may seriously impair the effectiveness of DOX chemotherapy [6]. In clinic treatment, DOX is chosen as the first-line drug and long-term use could result in drug resistance in low dosage [7]. Besides, the high dosage could result in toxic. DOX is a semi-synthetic of paclitaxel, which can promote the assembly of microtubules and inhibit their depolymerization, thus leading to cell cycle arrest and apoptosis. In in vitro cellular trials, the antitumor effect of DOX has been confirmed in OS cell lines. Therefore, investigation for DOX resistance in OS treatment is urgent for its clinical application.

Aerobic glycolysis, also known as Warburg effect, indicates the significant increased glycolysis level in tumor cells [8]. Tumor cells preferred to use aerobic glycolysis pathway for energy supply not only in hypoxia, but also in aerobic environment [9]. The "metabolic reprogramming" in tumor cells provides a new idea for tumor therapy [10]. The metabolic transformation from oxidative phosphorylation to glycolysis in tumor cells can affect the characteristics of tumor cells and participate in the regulation of tumor invasion and remote metastasis [11]. In OS, aerobic glycolysis significantly participates in the tumorigenesis [12]. For instance, S1P/S1PR3 axis promotes aerobic glycolysis in OS via inhibiting the phosphorylation of YAP and promoting YAP nuclear translocation of, which contributes to the YAP-c-MYC complex formation and enhances glycolysis enzyme PGAM1 transcription [13]. Thus, these data suggest that aerobic glycolysis remarkably regulates the OS progression.

In the present study, the novel circRNA circARHGAP12 regulates the metabolic progression in DOX-resistant OS cells. The novel circARHGAP12 (hsa_circ_0000231) has been reported as a m^6^A-modified circRNA in human cancer. CircARHGAP12 was generated from the exon-3 to exon-2 (794 bp length) of ARHGAP12 gene. Our research revealed that circARHGAP12 was significantly up-regulated in the OS cells and participated in the tumor chemoresistant and energetic metabolism.

## Materials and methods

### Cell culture and treatment

The human OS cell lines (Saos-2, MG63) and normal cells (hFOB) were purchased from the ATCC (American Type Culture Collection) and National Collection of Authenticated Cell Culture. The Saos-2 and MG63 cells were maintained in DMEM/F12 medium supplemented with FBS (10%, Biological Industries) and penicillin–streptomycin (1%) in a humidified incubator at 5% CO_2_ in 37℃. DOX-resistant OS cell lines were derived from original parental OS cell lines by continuous DOX exposure as previously described [14]. DOX-resistant OS cells were constructed in a dose-escalation manner (10, 25, 50, 100, 250 and 500 nM). The acquired drug resistance process took 7–8 months. In parallel, the parental OS cells were exposed to DMSO in the same dose-escalation manner, as vehicle solution.

### RNA extraction and real-time fluorescent quantitative polymerase chain reaction (RT-PCR)

Total RNA was extracted from OS cells using TRIzol cleavage agent (Invitrogen, Carlsbad, CA, USA). The RNA was then reversely transcribed into cDNA using the HiScript 1st Strand cDNA Synthesis Kit (Cat# 04897030001, Roche, USA). Quantitative polymerase chain reaction (qPCR) was performed using the Real-time Fluorescent Quantitative PCR Kit (Vazyme, Cat# Q321) using ABI7900HT Fast Real-Time PCR system (Applied Biosystems, CA, USA). Actin was used as endogenous control, and the comparative 2^−△△CT^ method was applied to calculate the relative expression. These primers were used and listed in Author contributions

### Transfection

The circARHGAP12 overexpression (OV) plasmids and human circARHGAP12 cDNA were synthesized and cloned into the pLC5-ciR vector (Geneseed, Fairfield, CT, USA). To knockdown the expression of circARHGAP12, oligonucleotides encoding short hairpin RNAs (shRNA) specific for circARHGAP12 were designed and synthesized by RiboBio Co., Ltd. (Guangzhou, China) and cloned into the GV112 lentivirus vector (GeneChem). In addition, OS cells were infected by lentivirus with Polybrene (8 mg/mL) and screened by puromycin (2 mg/mL, Sigma-Aldrich) for 2 weeks to obtain stable cell lines. The sequences of shRNA used in present study are listed in Additional file 1: Table S1.

### Identification of the circRNA

Genomic DNA (gDNA) was extracted from Saos-2/DOX and MG63/DOX cells using PureLink Genomic DNA Mini Kit (Invitrogen). After being amplified by convergent/divergent primers using cDNA and gDNA, the PCR products of RNA were detected, including circRNA, β-actin and ARHGAP12. In RNase R assay, the extracted RNAs from Saos-2/DOX and MG63/DOX cells were incubated with or without RNase R, and then, RNA was used for detecting circARHGAP12 expression and linear ARHGAP12 expression.

### DOX sensitivity assay

The DOX resistance was determined by IC50 value (50% maximal inhibitory concentration) using CCK-8 assay. After the cellular transfection and DOX treatment, OS cells (5 × 10^3^ cells/well) were seeded into 96-well plate and their cellular viability was detected every 24 h for 4 days. CCK-8 reagent (10 µL, Solarbio Science, Beijing, Cat no. #CA1210) was added to each well. After incubation and maintain for 1.5 h at 37 °C, the absorbance of each well was measured at 450 nm using spectrophotometer.

### Glucose, lactate, and ATP analysis assay

For the glucose uptake assay, the transfected cells were harvested for measurement of glucose concentrations using glucose assay kit (Sigma, St-Louis, MO, USA) according to the manufacturer’s instructions. For lactate production assay, the lactate concentration in the OS cells’ lysis was detected using lactate assay kit (K627, BioVision) according to the manufacturer’s instructions. For ATP analysis, ATP assay kit (S0026,Beyo time) was performed to detect intracellular ATP in cellular extracts by detecting the luciferase activity.

### Extracellular acidification assays

To detect the extracellular acidification rate (ECAR), OS cells (1 × 10^4^ cells/well) were seeded into Seahorse XF 96 cell culture microplates. After adhere overnight, cells were washed using DMEM medium supplemented with L-glutamine (2 mM, pH = 7.4) for incubation at 37 °C in CO_2_-free incubator. Then, the microplates were loaded into the Seahorse Analyzer including mixture (4.5 min), wait (0 min), and measure (4.5 min) for 3 cycles. OS cells were sequentially administrated with glucose (10 mM), the oxidative phosphorylation inhibitor oligomycin (1.0 μM), and the glycolytic inhibitor 2-deoxyglucose (2-DG, 50 mM) in each well at indicated time points. The ECAR was performed using the Seahorse XF 96 Extracellular Flux Analyzer (Agilent Technologies, Santa Clara, CA, USA) according to the manufacturer’s instructions.

### Colony formation assay

The OS cells (Saos-2/Dox, MG63/Dox) were transfected with circARHGAP12 overexpression and knockdown and then placed in fresh 6-well plates and maintained in RPMI-1640 medium containing FBS (10%, HyClone, Logan, UT, USA). After two weeks, OS cells were fixed with methanol and then stained with crystal violet (0.1%, Beyotime Institute of Biotechnology). Visible clone was manually counted based on five random visual field.

### m^6^A RIP-RT-qPCR analysis

To detect the m^6^A modification levels of c-Myc mRNA, m^6^A RIP assay was performed using Magna MeRIP m^6^A kit (Millipore Sigma, Billerica, Cat no. MA17-10499) according to manufacturer’s instruction. In brief, the isolated RNA was fragmented using fragmentation buffer. Part of total RNA acted as input, RNA was immunoprecipitated to m^6^A antibody-conjugated magnetic beads. After being washing with immunoprecipitation buffer, the m^6^A-modified c-Myc mRNAs were eluted with elution buffer. The RNA was subjected to qRT-PCR assays for c-Myc m^6^A modification sites quantitative analysis normalized to input.

### mRNA stability assay

For the mRNA stability analysis, OS cells were cultured in completed medium containing Act-D (5 μg/mL Sigma-Aldrich) to block the transcription of c-Myc mRNA. After incubation for 0/3/6 h, total RNA was extracted from OS cells and the c-Myc mRNA expression level was then detected at indicated time point.

### Statistical analysis

All experiments were performed in triplicate and data were presented as Mean ± SD. All the average value was calculated based on independent experiments. GraphPad Prism 8.0 and SPSS 20.0 software were utilized to analyze the standard deviation. Statistical analysis was calculated by Student’s t test or ANOVA. *P* < 0.05 was considered as significance.

## Results

### circARHGAP12 was a DOX-resistant circRNA in OS

**Fig. 1:**
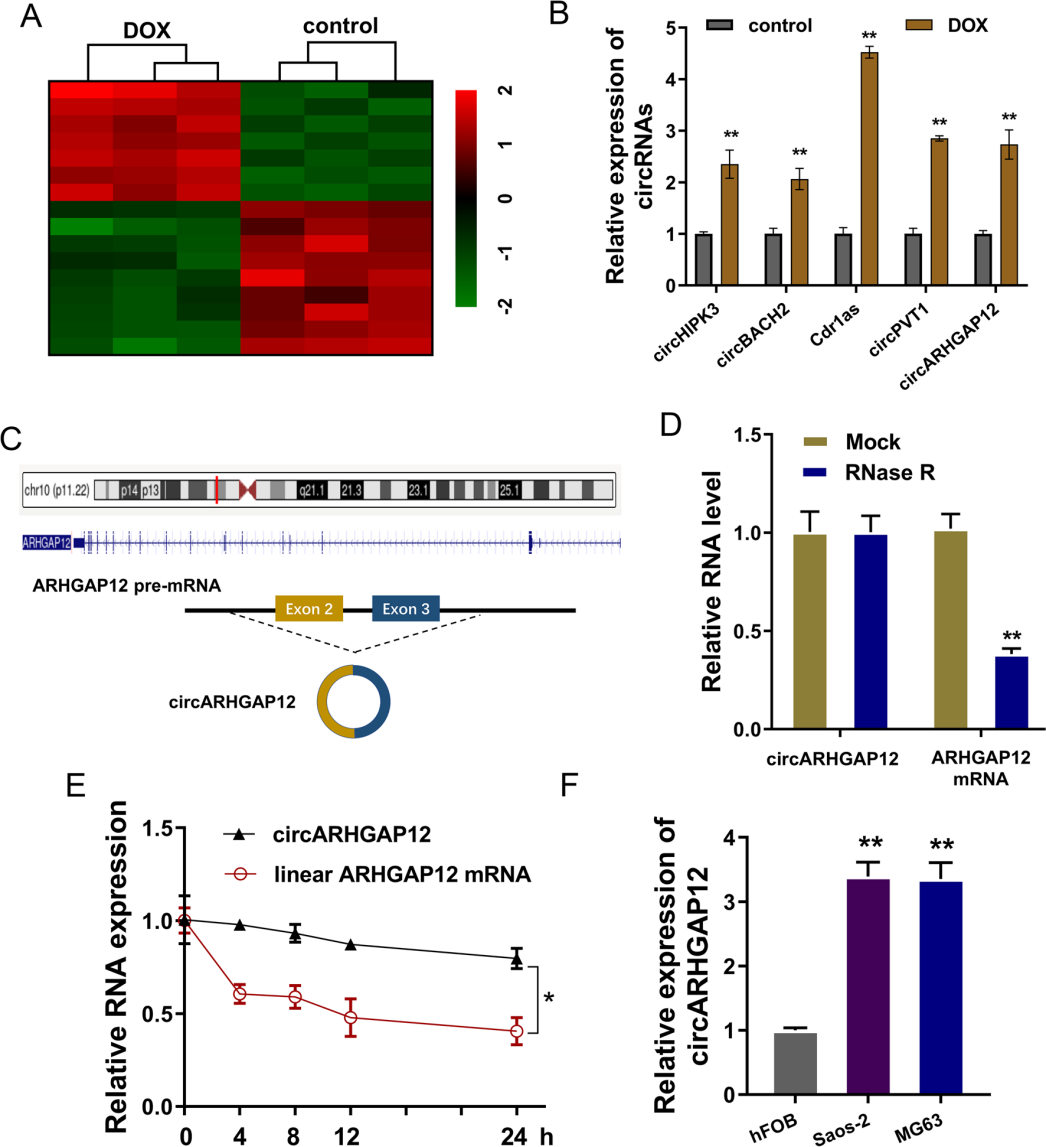
circARHGAP12 was a DOX-resistant circRNA in OS. **A** CircRNA microarray was performed to test the circRNA profile in the DOX-resistant OS cells (MG63/Dox) and parental cells (MG63). **B** RT-PCR was performed to test several potential circRNAs (circHIPK3, circBACH2, Cdr1as, circPVT1, circARHGAP12) in the DOX-resistant OS cells (MG63/Dox) and parental cells (MG63). **C** Schematic diagram illustrated the biogenesis of circARHGAP12 (circARHGAP12, hsa_circ_0000231) generated from the exon-3 to exon-2 (794 bp length) of ARHGAP12 gene. **D** RNase R assay was performed to detect the stability of circARHGAP12 in DOX-resistant OS cells (MG63/Dox), comparing with liner transcript ARHGAP12 mRNA. **E** Actinomycin D assay was performed to detect the stability of circARHGAP12 in DOX-resistant OS cells (MG63/Dox). (**F**) RT-PCR was performed to detect the circARHGAP12 in OS cells (Saos-2, MG63) compared to normal cells (hFOB). Data were presented as means ± SD from three independent experiments. *p < 0.05, **p < 0.01

To investigate the differentially expressed circRNA in the DOX-resistant OS, our work performed the circRNA microarray to test the circRNA profile. Results indicated that there were numerous differential circRNAs in the DOX-resistant OS cells (MG63/Dox) and parental cells (MG63) (Fig. 1A). Then, our work tested several potential circRNAs (circHIPK3, circBACH2, Cdr1as, circPVT1, circARHGAP12) in the DOX-resistant OS cells (MG63/Dox) and parental cells (MG63), showing the high-expression of these candidate circRNAs (Fig. 1B). From these circRNAs, our study selected the circARHGAP12 for further research. CircARHGAP12 was m^6^A-modified circRNA [15] and generated from the exon-3 to exon-2 (794 bp length) of ARHGAP12 gene. To detect the stability of circARHGAP12, RNase R assay administration (Fig. 1D) and Actinomycin D treatment (Fig. 1E) were performed in DOX-resistant OS cells (MG63/Dox). Results indicated that circARHGAP12 was more stable than its linear transcript (ARHGAP12 mRNA) when administrated with extraneous interposition. In OS cells (Saos-2, MG63), circARHGAP12 was remarkably up-regulated in the OS cells compared to normal cells (Fig. 1F). Taken together, these data indicated that circARHGAP12 was a DOX-resistant circRNA in OS.

### circARHGAP12 accelerated the Dox-resistance and proliferation of OS cells

**Fig. 2:**
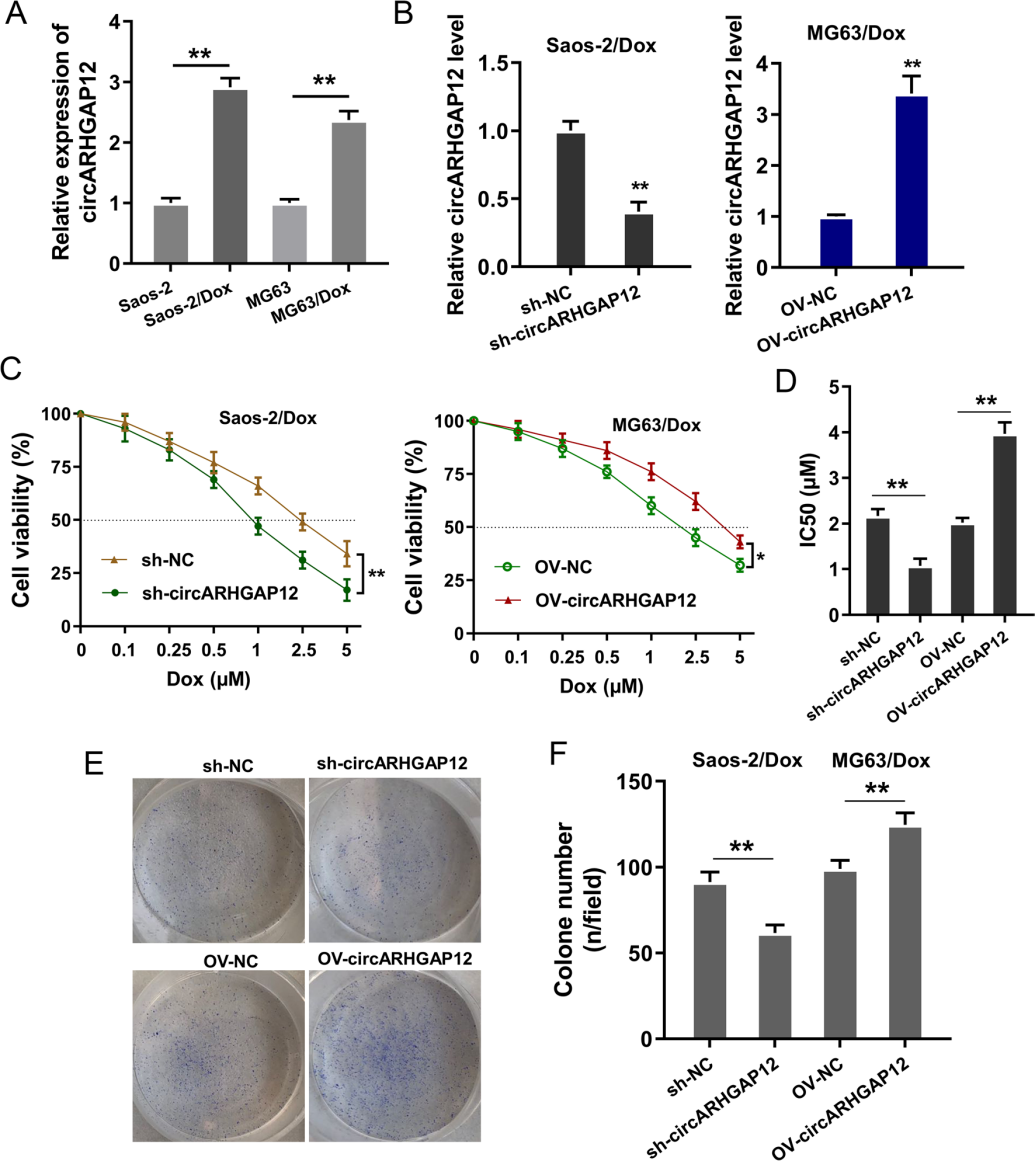
circARHGAP12 accelerated the Dox-resistance and proliferation of OS cells. **A** RT-PCR assay was performed to detect the circARHGAP12 level in DOX-resistant OS cells (Saos-2/Dox, MG63/Dox) and parental cells (Saos-2, MG63). **B** The silencing and overexpression of circARHGAP12 were constructed and, respectively, transfected into Dox-resistant OS cells (Saos-2/Dox, MG63/Dox) for gain/loss function assays. **C**, **D** The Dox resistance of OS cells (Saos-2/Dox, MG63/Dox) was determined by IC50 value (50% maximal inhibitory concentration) using CCK-8 assay. **E**, **F** Clone formation assay was performed to detect the colony number of Saos-2/Dox cells with circARHGAP12 silencing, and MG63/Dox cells with circARHGAP12 overexpression. Data were presented as means ± SD from three independent experiments. **p < 0.01, *p < 0.05

Given that our findings showed the doxorubicin (Dox)-resistant character for circARHGAP12 in OS cells, our study tests the function of circARHGAP12 on OS cells. Dox acts as the standard treatment approach for OS, while acquired drug resistance seriously attenuates the clinical treatment efficiency. Firstly, the level of circARHGAP12 was much higher in DOX-resistant OS cells (Saos-2/Dox, MG63/Dox) and parental cells (Saos-2, MG63) (Fig. 2A). The silencing and overexpression of circARHGAP12 were constructed and, respectively, transfected into Dox-resistant OS cells (Saos-2/Dox, MG63/Dox) for further assay (Fig. 2B). The Dox-resistance using CCK-8 assay revealed that circARHGAP12 silencing inhibited the IC50 value (50% maximal inhibitory concentration) of Dox in Saos-2/Dox, and circARHGAP12 overexpression up-regulated the IC50 value of Dox in MG63/Dox (Fig. 2C, D). Clone formation assay revealed that circARHGAP12 silencing inhibited the colony number of Saos-2/Dox cells, and circARHGAP12 overexpression promoted the colony number of MG63/Dox cells (Fig. 2E, F). Taken together, these data indicated that circARHGAP12 accelerated the Dox-resistance and proliferation of OS cells.

### circARHGAP12 promoted the aerobic glycolysis of OS cells

**Fig. 3:**
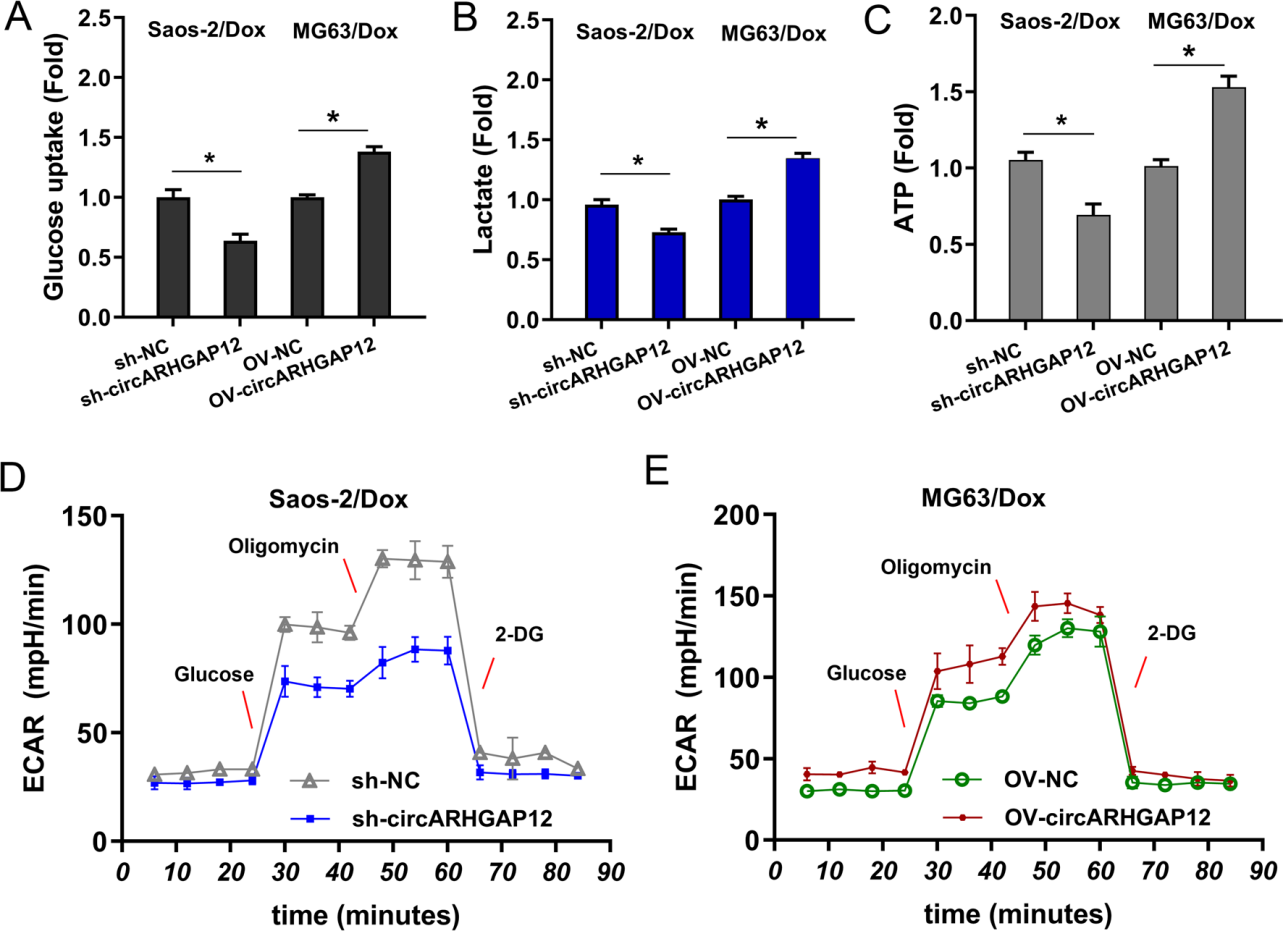
circARHGAP12 promoted the aerobic glycolysis of OS cells. **A** The glucose uptake level analysis was performed to detect the glucose uptake of Saos-2/Dox cells with circARHGAP12 silencing, and MG63/Dox cells with circARHGAP12 overexpression. **B** Lactate production was performed to detect the lactate level of Saos-2/Dox cells with circARHGAP12 silencing, and MG63/Dox cells with circARHGAP12 overexpression. **C** ATP analysis was performed to detect the ATP level of Saos-2/Dox cells with circARHGAP12 silencing, and MG63/Dox cells with circARHGAP12 overexpression. **D**, **E** Metabolic flux analysis was performed to detect the glycolysis rate of Saos-2/Dox and MG63/Dox cells with circARHGAP12 overexpression/silencing for extracellular acidification rate (ECAR). Data were presented as means ± SD from three independent experiments. *p < 0.05

The effect of glycolysis upon chemotherapy resistance is relatively unclear. Emerging research has revealed that aerobic glycolysis participated in the chemotherapy resistance, thus this present study tried to investigate the function of circARHGAP12 on aerobic glycolysis of OS cells. Results indicated that circARHGAP12 silencing repressed the glucose uptake (Fig. 3A), lactate production (Fig. 3B), ATP generation (Fig. 3C) and extracellular acidification rate (ECAR) (Fig. 3D, E). Besides, circARHGAP12 overexpression promoted the glucose uptake, lactate production, ATP generation and extracellular acidification rate (ECAR). These findings revealed that circARHGAP12 positively promoted the aerobic glycolysis of OS cells.

### c-Myc acted as a m^6^A-modified target in OS cells

**Fig. 4:**
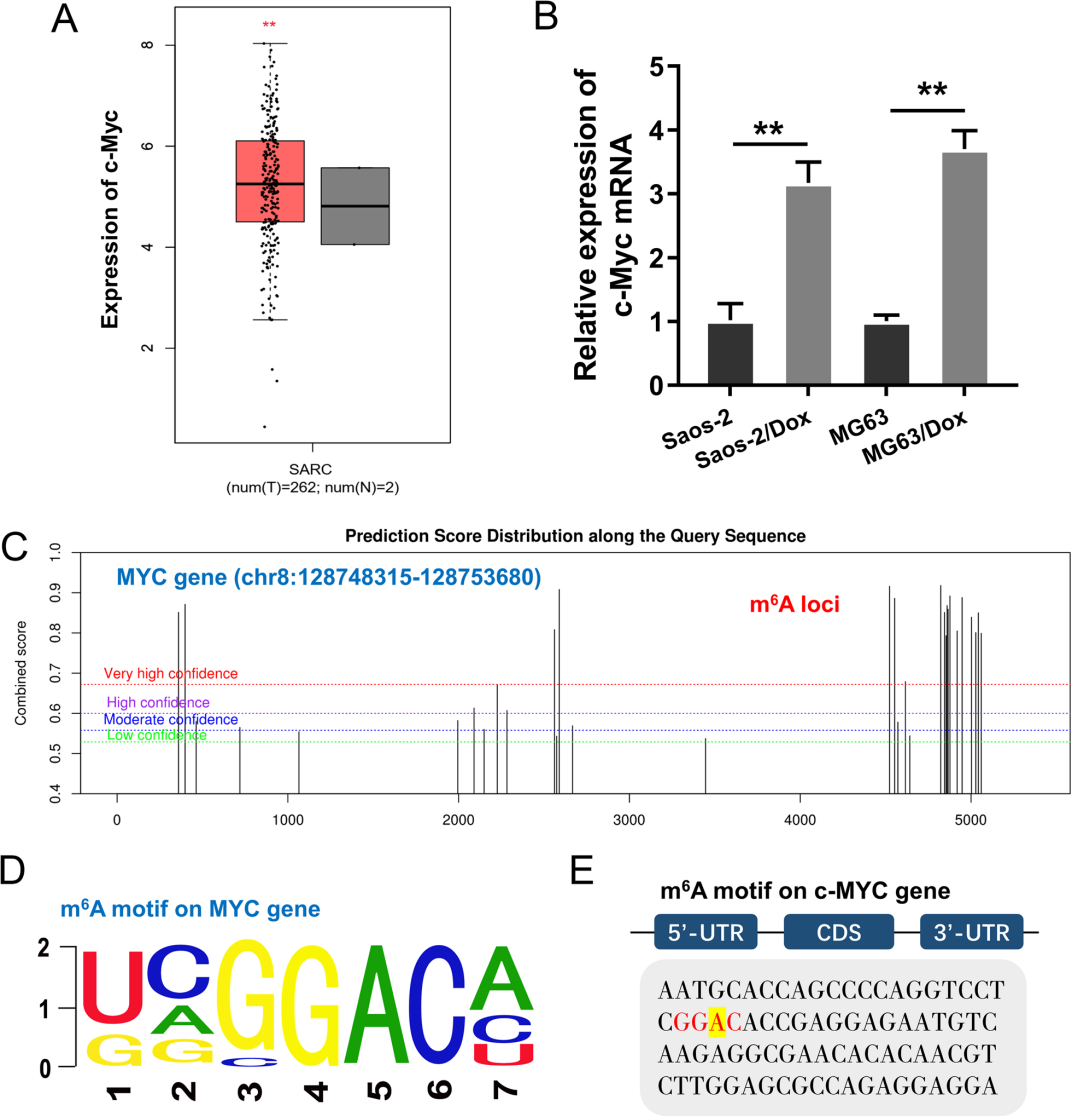
c-Myc acted as a m^6^A-modified target in OS. **A** In silico tools analysis (http://gepia.cancer-pku.cn/index.html) showed the up-regulated level in the osteosarcoma (SARC) samples. **B** RT-PCR was performed to test the level of c-Myc in DOX-resistant OS cells (Saos-2/Dox, MG63/Dox) and parental cells (Saos-2, MG63). **C** In silico predictive analysis (http://www.cuilab.cn/sramp)showed that there were several potential m^6^A modified sites in the c-Myc gene. **D** m^6^A motif predicted by in silico tools for c-Myc gene (GGAC). **E** The accurate loci on c-Myc gene (GGAC). Data were presented as means ± SD from three independent experiments. **p < 0.01

Given that circARHGAP12 regulated the OS cells’ aerobic glycolysis, our study tried to investigate the potential target of circARHGAP12 in OS cells. Firstly, the c-Myc level significantly up-regulated in the osteosarcoma samples (Fig. 4A). Firstly, the level of c-Myc was higher in DOX-resistant OS cells (Saos-2/Dox, MG63/Dox) and parental cells (Saos-2, MG63) (Fig. 4B). In silico predictive analysis (http://www.cuilab.cn/sramp)showed that there were several potential m^6^A modified sites in the c-Myc genome (Fig. 4C). The m^6^A motif predicted by in silico tools for the c-Myc gene was GGAC (Fig. 4D). The accurate loci of c-Myc gene were identified as GGAC (Fig. 4E). These findings revealed that c-Myc acted as a m^6^A-modified target in OS cells.

### m^6^A-modified circARHGAP12 enhanced the stability of c-Myc mRNA via m^6^A-dependent manner

**Fig. 5:**
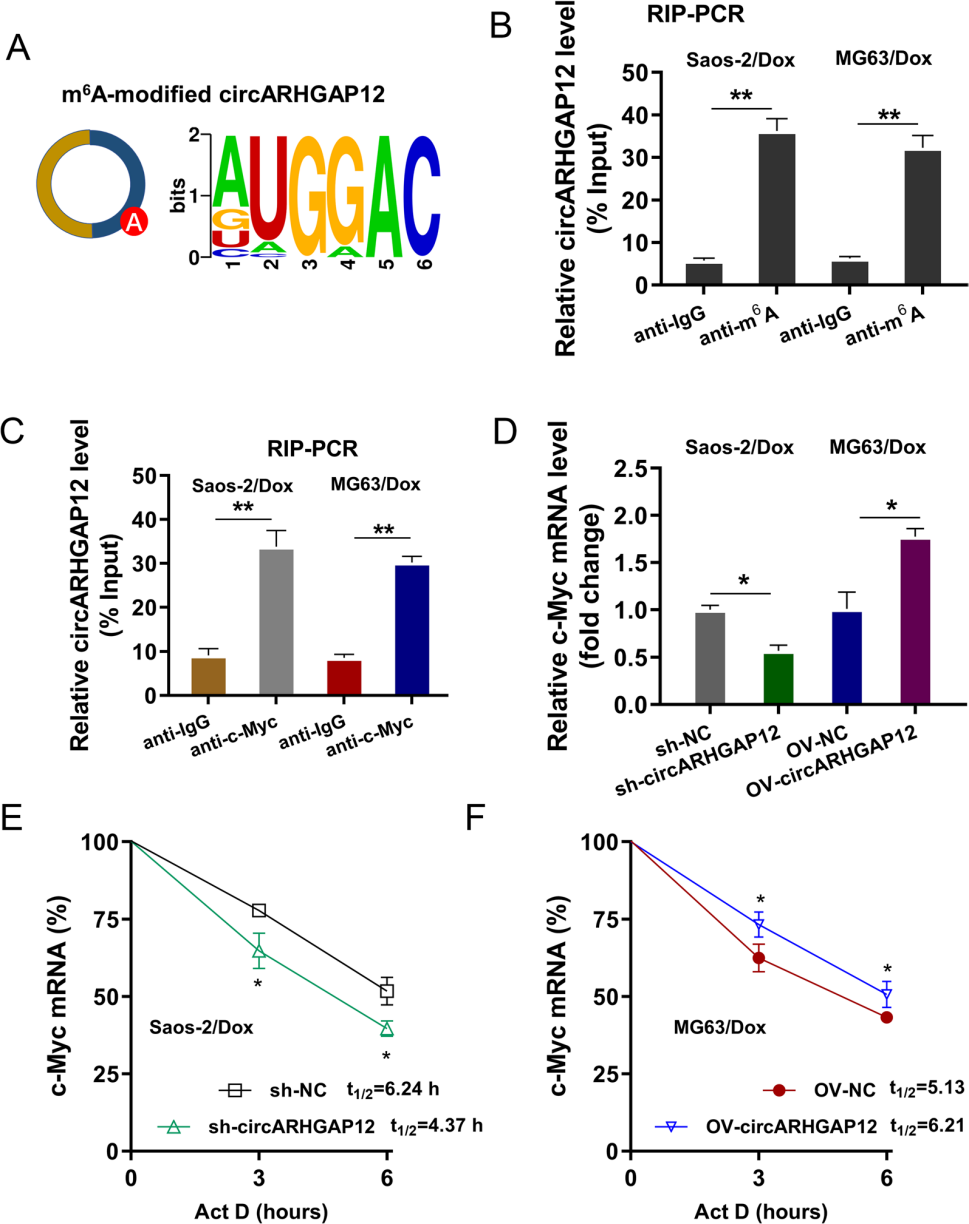
m^6^A-modified circARHGAP12 enhanced the stability of c-Myc mRNA via m^6^A-dependent manner. **A** There was a remarkable m^6^A-modified loci in the circARHGAP12. **B** The RIP-PCR assay using the anti-m^6^A antibody was conducted in DOX-resistant OS cells (Saos-2/Dox, MG63/Dox). The circARHGAP12 level was tested in the immunoprecipitation of anti-m^6^A antibody. **C** The RIP-PCR assay using the anti-c-Myc antibody was conducted in DOX-resistant OS cells (Saos-2/Dox, MG63/Dox). The circARHGAP12 level was tested in the immunoprecipitation of anti-c-Myc antibody. **D** The RT-PCR assay was conducted in DOX-resistant OS cells (Saos-2/Dox, MG63/Dox). The c-Myc mRNA level was tested. **E**, **F** RNA stability analysis using Act D was conducted in DOX-resistant OS cells (Saos-2/Dox, MG63/Dox). The half-life time (t_1/2_) of c-Myc mRNA was calculated for c-Myc mRNA stability. Data were presented as means ± SD from three independent experiments. **p < 0.01, *p < 0.05

**Fig. 6:**
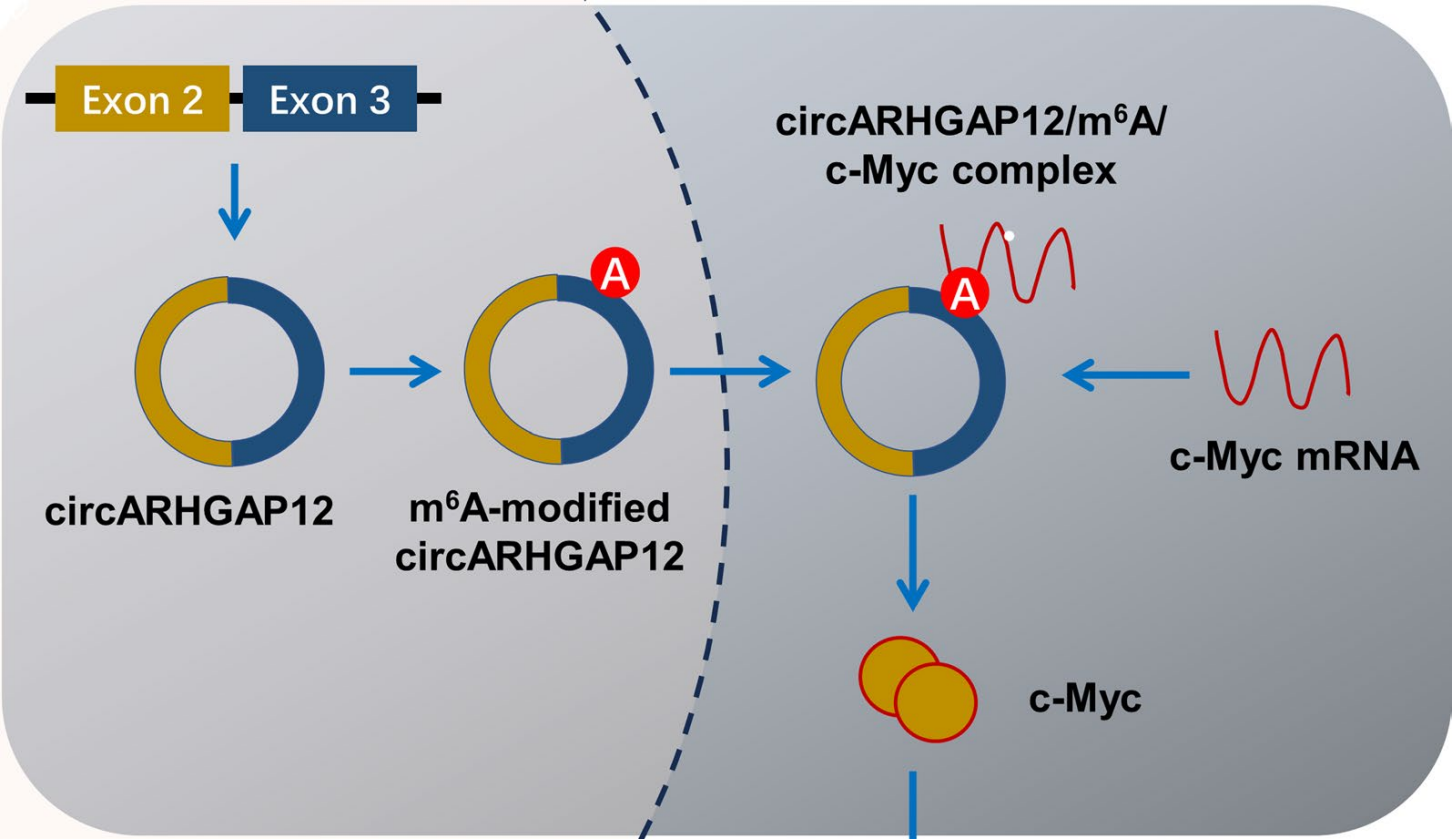
m^6^A-modified circARHGAP12 promoted the aerobic glycolysis of doxorubicin-resistance osteosarcoma through m^6^A/c-Myc manner

The novel circARHGAP12 (hsa_circ_0000231) has been reported as a m^6^A-modified circRNA in human cancer [15]. There was a remarkable m^6^A-modified loci in the circARHGAP12 (Fig. 5A). Using the anti-m^6^A antibody, the RIP assay revealed that circARHGAP12 significantly combined with anti-m^6^A antibody in DOX-resistant OS cells (Saos-2/Dox, MG63/Dox) (Fig. 5B). Moreover, to test whether circARHGAP12 could combine with the c-Myc, the RIP assay was performed using anti-c-Myc antibody. Results indicated that circARHGAP12 significantly connected with c-Myc in DOX-resistant OS cells (Fig. 5C). In DOX-resistant OS cells, circARHGAP12 silencing repressed the c-Myc mRNA level. Besides, circARHGAP12 overexpression promoted the c-Myc mRNA level (Fig. 5D). RNA stability analysis using Act D indicated that circARHGAP12 silencing repressed the half-life time (t_1/2_) of c-Myc mRNA, and circARHGAP12 overexpression up-regulated the half-life time (t_1/2_) of c-Myc mRNA, indicating the positive role of circARHGAP12 on c-Myc mRNA stability (Fig. 5E, F). Taken together, these findings inspired that m^6^A-modified circARHGAP12 enhanced the stability of c-Myc mRNA via m^6^A-dependent manner (Fig. 6).

## Discussion

Doxorubicin (Dox) functions as the standard treatment approach for OS treatment; however, acquired drug resistance earnestly moderate the treatment efficiency [16]. It has been indicated that tumor metabolic processes, such as aerobic glycolysis, always shift the chemoresistant tumor cells. The tumor chemoresistance and energy metabolism could prominently regulate the OS tumorigenesis.

The effect of circRNA and m^6^A methylation on accelerating OS chemoresistant is also investigating [17, 18]. Here, our study tried to detect the expression, function and mechanism of m^6^A-modified circARHGAP12 in OS cells. Dox-resistance analysis revealed that circARHGAP12 silencing inhibited the IC50 value (50% maximal inhibitory concentration) of Dox in Saos-2/Dox, and circARHGAP12 overexpression up-regulated the IC50 value of Dox in MG63/Dox, indicating that circARHGAP12 accelerated the Dox-resistant OS cells. Chemotherapy resistance is a major obstacle to tumor treatment [19]. The doxorubicin functions as the standard treatment approach for OS; however, acquired drug resistance earnestly moderate the treatment efficiency.

Given the treatment dilemma of acquired drug resistance, the in-depth mechanism investigation for chemotherapy resistance is attractive and prospective for solving the hard problem. Therefore, herein, the metabolic potential between chemo-resistant OS cells was compared. Here, we investigate the potential etiological factors for chemotherapy resistance. The effect of glycolysis upon chemotherapy resistance is relatively unclear. Emerging research has revealed that aerobic glycolysis participated in the chemotherapy resistance, and thus, this present study tried to investigate the function of circARHGAP12 on aerobic glycolysis of OS cells. We found that circARHGAP12 silencing repressed the glucose uptake, lactate production, ATP generation and extracellular acidification rate (ECAR). Besides, circARHGAP12 overexpression promoted the glucose uptake, lactate production, ATP generation and ECAR. Thus, findings revealed that circARHGAP12 positively promoted the aerobic glycolysis of OS cells. Aerobic glycolysis was found to be closely correlated with OS chemotherapy resistance.

The function of circRNA and m^6^A methylation on accelerating tumor chemoresistance and aerobic glycolysis is emerging important [20]. Researches have illustrated that many elements regulate the chemoresistance, such as ATP generation, glucose consumption, lactate production and ECAR. For example, circARHGAP29 triggers the docetaxel resistance and aerobic glycolysis in prostate cancer cells, and circARHGAP29 increases the lactate dehydrogenase A (LDHA) mRNA stability of strengthening its interaction to insulin-like growth factor 2 mRNA-binding protein 2, resulting in enhanced glycolytic metabolism [21]. Circular RNA hsa_circ_0005963 (termed ciRS-122) sponges miR-122 for PKM2-targeting and positively correlates with chemoresistance, thereby promoting CRC chemoresistant cells glycolysis and drug resistance [22].

In present research, the findings reveal that circARHGAP12 is a m^6^A-modified circRNA in doxorubicin-resistance OS cells. circARHGAP29 interacts with c-Myc mRNA and stabilizes c-Myc mRNA, which further promotes c-Myc expression by enhancing its transcription. CircARHGAP12 promotes the aerobic glycolysis of dox-resistance OS through m^6^A/c-Myc manner, providing a promising therapeutic target for Dox-resistant OS.

### Supplementary Information

**Additional file 1.** **Table S1**. Primer sequences.

## Data Availability

Not applicable.
