# Supplementary material for: m6A-modified circARHGAP12 promotes the aerobic glycolysis of doxorubicin-resistance osteosarcoma by targeting c-Myc
Source: J Orthop Surg Res. 2024 Jan 4;19:33. doi: 10.1186/s13018-023-04502-0 (PMC10768094; doi:10.1186/s13018-023-04502-0)
Supplement: Supplementary file 1 — Additional file 1. Table S1. Primer sequences. [file 13018_2023_4502_MOESM1_ESM.docx]

**Supplement Table 1**. Primers sequences.

|  | Sequences |
| --- | --- |
| circARHGAP12 | forward, 5’-ATCTTGTGATTCCGCAGGAG-3’  reverse, 5’-ATGGCTTTATGGCTTGTTGG-3’ |
| sh-circARHGAP12 | 5’-CACTGAACAGATAAGGGTTTA-3’ |
| c-Myc | forward, 5’- GGCTCCTGGCAAAAGGTCA-3’  reverse, 5’- CTGCGTAGTTGTGCTGATGT-3’ |
| Beta-actin | forward, 5’-CTCCTTAATGTCACGCAGGATTTC-3’  reverse, 5’-GTGGGGCGCCCCAGGCACCA-3’ |
| GAPDH | forward, 5’-GGTATGACAACGAATTTGGC-3’  reverse, 5’-GAGCACAGGGTACTTTATTG-3’ |
